# Aging-Associated Dysfunction of Akt/Protein Kinase B: S-Nitrosylation and Acetaminophen Intervention

**DOI:** 10.1371/journal.pone.0006430

**Published:** 2009-07-29

**Authors:** Miaozong Wu, Anjaiah Katta, Murali K. Gadde, Hua Liu, Sunil K. Kakarla, Jacqueline Fannin, Satyanarayana Paturi, Ravi K. Arvapalli, Kevin M. Rice, Yeling Wang, Eric R. Blough

**Affiliations:** 1 Department of Biological Sciences, Marshall University, Huntington, West Virginia, United States of America; 2 Cell Differentiation and Development Center, Marshall University, Huntington, West Virginia, United States of America; 3 Department of Pharmacology, Physiology and Toxicology, Marshall University, Huntington, West Virginia, United States of America; 4 Department of Exercise Science, Sport and Recreation, Marshall University, Huntington, West Virginia, United States of America; 5 Department of Physiology and Pharmacology, Southeast University, Nanjing, China; 6 The First Hospital, Jilin University, Jilin, China; University of Valencia, Spain

## Abstract

**Background:**

Aged skeletal muscle is characterized by an increased incidence of metabolic and functional disorders, which if allowed to proceed unchecked can lead to increased morbidity and mortality. The mechanism(s) underlying the development of these disorders in aging skeletal muscle are not well understood. Protein kinase B (Akt/PKB) is an important regulator of cellular metabolism and survival, but it is unclear if aged muscle exhibits alterations in Akt function. Here we report a novel dysfunction of Akt in aging muscle, which may relate to S-nitrosylation and can be prevented by acetaminophen intervention.

**Principal Findings:**

Compared to 6- and 27-month rats, the phosphorylation of Akt (Ser473 and Thr308) was higher in soleus muscles of very aged rats (33-months). Paradoxically, these increases in Akt phosphorylation were associated with diminished mammalian target of rapamycin (mTOR) phosphorylation, along with decreased levels of insulin receptor beta (IR-β), phosphoinositide 3-kinase (PI3K), phosphatase and tensin homolog deleted on chromosome 10 (PTEN) and phosphorylation of phosphoinositide-dependent kinase-1 (PDK1) (Ser241). *In vitro* Akt kinase measurements and *ex vivo* muscle incubation experiments demonstrated age-related impairments of Akt kinase activity, which were associated with increases in Akt S-nitrosylation and inducible nitric oxide synthase (iNOS). Impairments in Akt function occurred parallel to increases in myocyte apoptosis and decreases in myocyte size and the expression of myosin and actin. These age-related disorders were attenuated by treating aged (27-month) animals with acetaminophen (30 mg/kg body weight/day) for 6-months.

**Conclusions:**

These data demonstrate that Akt dysfunction and increased S-nitrosylation of Akt may contribute to age-associated disorders in skeletal muscle and that acetaminophen may be efficacious for the treatment of age-related muscle dysfunction.

## Introduction

Decreases in muscle size and strength, diminished protein synthesis and an increased incidence of muscle cell apoptosis are well characterized aspects of aging in both human and animal models [1]–[3]. The deleterious effects of aging appear to accelerate over time and are important contributors to increased frailty and mortality in the aged [2], [4]. The cellular mechanism(s) that are responsible for these changes are not well understood and have not been widely studied.

Protein kinase B (Akt/PKB) is a serine-threonine protein kinase that plays a central role in integrating anabolic and catabolic responses by transducing the signals emanating from growth factors, nutrients, cytokines and muscle contraction via changes in the phosphorylation of its numerous substrates [5]–[9]. Activation of Akt stimulates protein synthesis, muscle hypertrophy and cell survival while it antagonizes the loss of muscle protein [6], [7]. Given the multifunctional roles ascribed to Akt it is likely that this molecule could play a critical role in mediating aging-associated disorders in cellular metabolism and physiological function. Although alterations in Akt abundance and phosphorylation have been shown in aging muscle [5], [6], little is known about whether or not aging affects Akt kinase function. Akt function is controlled, at least in part, by the phosphorylation of Ser473 by the mammalian target of rapamycin (mTOR) and the phosphorylation of Thr308 by phosphoinositide-dependent kinase (PDK)-1 [10]–[12]. In addition to these positive regulators, Akt signaling can also be negatively regulated by the phosphatase and tensin homolog deleted on chromosome 10 (PTEN) [13], [14], S-nitrosylation induced by increases in nitric oxide (NO) [15]–[20] and elevated extracellular glucose [21], [22]. Whether age-related alterations in the amount or regulation of these factors affect Akt function in aging skeletal muscle is not known.

The purpose of this study was to examine if aging-related changes in skeletal muscle structure are associated with alterations in Akt function. On the basis of previous work demonstrating that chronic acetaminophen (*N*-acetyl *p*-aminophenol, APAP) intake (30 mg/kg body weight/day) can be safely (e.g. in the absence of hepatotoxicity) used to prevent age-associated hyperglycemia [23], and other findings suggesting that elevated glucose levels can induce iNOS expression [19], [20] while reduce PTEN expression [14], we tested if this type of treatment regimen would also be effective in improving Akt function. We hypothesized that aging would be associated with impairment of Akt kinase function, increases in muscle apoptosis and atrophy, and that these deficits could be ameliorated, at least in part, by chronic acetaminophen treatment. To test these possibilities, we examined the relationship between Akt function, the S-nitrosylation of Akt, and soleus muscle apoptosis and atrophy in very aged (33-month old) Fischer344/NNiaHSD×Brown Norway/BiNia (F344BN) rats that had been daily treated with acetaminophen (30 mg/kg body weight/day) for 6 months. Our results show that aging skeletal muscle exhibits impaired Akt kinase activity and that acetaminophen-induced improvements in Akt signaling are associated with increases in myocyte size and the expression of myosin and actin, along with decreases in muscle apoptosis. Given the economic significance of an aging population on society and its health system, these data provide evidence that improving Akt function may be a useful strategy for improving muscle structure and suggest that acetaminophen may be efficacious for the treatment of age-related muscle dysfunction.

## Results

### Aging-associated hyper-phosphorylation of Akt can be attenuated by chronic acetaminophen treatment

**Figure 1:**
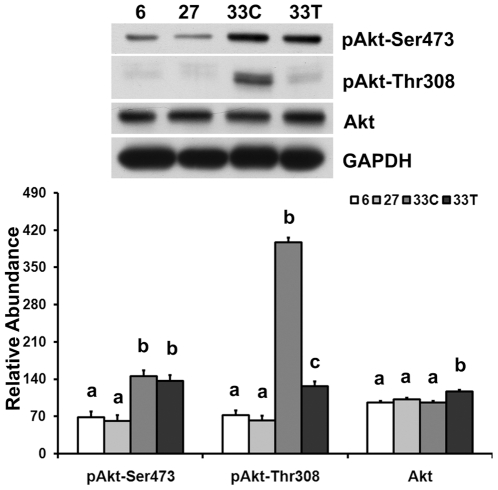
Aging-associated hyper-phosphorylation of Akt can be attenuated by acetaminophen. Akt total protein and the phosphorylation of Akt at Ser473 and Thr308 in soleus muscle from 6-, 27-, 33-month control (33C) and acetaminophen-treated (33T) F344BN rats were determined by immunoblotting. Data are mean±SE (n = 4–6). abc: Groups without the same letter are significantly different (P<0.05).

Compared to that observed in adult animals (6-month), the phosphorylation of Akt at the Ser473 was increased by 114.3% in the soleus of 33-month aged rats (P<0.05; Figure 1). Acetaminophen treatment did not alter the abundance of pAkt-Ser473 compared to that found in age-matched controls (P>0.05). Phosphorylation of Akt at the Thr308 was increased by 450.9% in 33-month control animals (P<0.05), while chronic acetaminophen treatment significantly decreased the abundance of pAkt-Thr308 (−68.1%, P<0.05).

### Akt hyper-phosphorylation does not result in increased phosphorylation of the Akt downstream molecules

**Figure 2:**
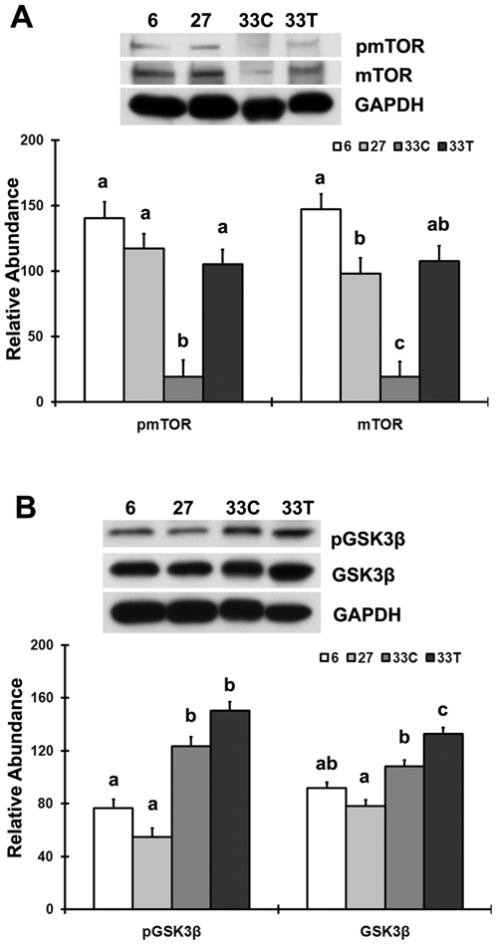
Akt hyper-phosphorylation does not result in increased phosphorylation of its downstream molecules. A. mTOR total protein and phosphorylation of mTOR at Ser2448 in 6-, 27-, 33-month control (33C) and acetaminophen-treated (33T) rats as determined by immunoblotting. B. GSK3β total protein and phosphorylated GSK3β (Ser9). Data are mean±SE (n = 4). abc: Groups without the same letter are significantly different (P<0.05).

The abundance of phosphorylated mTOR (pmTOR) (Ser2448) and mTOR total protein in the very aged soleus were lower than that in the adult animals (−86.3% and −86.8%, respectively; P<0.05; Figure 2A), while chronic acetaminophen treatment restored the amount of phosphorylated and total mTOR to a level equivalent to that seen in 6- and 27-month old animals (P>0.05). The ratio of pmTOR/mTOR was not different between age-matched controls and acetaminophen-treated rats (P>0.05).

The abundance of phosphorylated glycogen synthase kinase 3β (pGSK3β) (Ser9) was 61.7% higher in the very age rats (P<0.05; Figure 2B), and acetaminophen treatment trended to increase pGSK3β abundance compared to that observed in the age-matched control group (P = 0.06). Acetaminophen administration increased the abundance of total GSK3β by 22.4% when compared to that found in the age-matched controls (P<0.05). The ratio of pGSK3β/GSK3β was not different between age-matched controls and acetaminophen-treated rats (P>0.05).

### Akt hyper-phosphorylation does not appear to be related to increased insulin receptor beta (IR-β), phosphoinositide 3-kinase p85 (PI3K-p85) and PDK1

**Figure 3:**
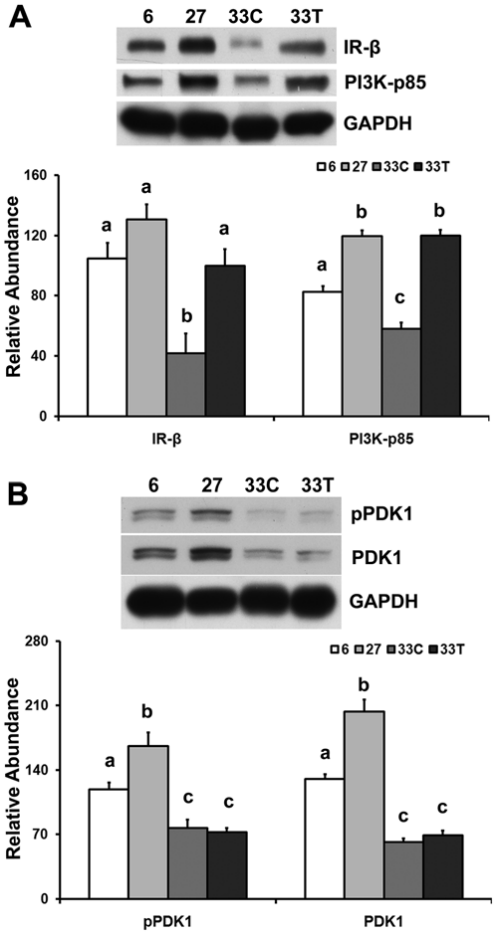
Akt hyper-phosphorylation does not appear to be related to increased IR-β, PI3K-p85, and PDK1 protein levels. A. IR-β and PI3K-p85 protein in 6-, 27-, 33-month control (33C) and acetaminophen-treated (33T) rats were determined by immunoblotting. B. PDK1 total protein and phosphorylation of PDK1 at Ser241 as determined by immunoblotting. Data are mean±SE (n = 4–6). abc: Groups without the same letter are significantly different (P<0.05).

Compared to adult animals, the abundance of IR-β and PI3K-p85 protein were lower in the soleus muscle of very aged control rats (−60.0% and −29.7%, respectively; P<0.05; Figure 3A). After 6 months of acetaminophen treatment, IR-β and PI3K-p85 protein abundances were restored to the level found in 27-month old animals (P>0.05).

The abundance of phosphorylated PDK1 (pPDK1) (Ser241) and PDK1 total protein in the very aged soleus were lower than that found in the adult animals (−35.2% and −52.5%, respectively; P<0.05; Figure 3B), while neither pPDK1 nor PDK1 were different between age-matched controls and acetaminophen-treated rats (P>0.05). The ratio of pPDK1/PDK1 was also not different between acetaminophen-treated and age-matched control animals (P>0.05).

### Dysfunction of Akt in the very aged muscle can be corrected by acetaminophen

**Figure 4:**
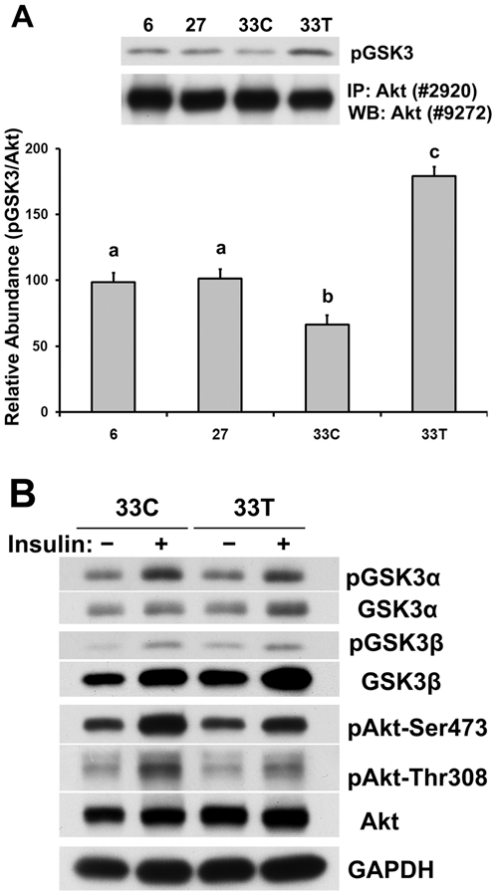
Akt dysfunction in the very aged soleus muscle can be corrected by acetaminophen. A. Akt kinase activity as measured in soleus muscles from 6-, 27-, 33-month control (33C) and acetaminophen-treated (33T) rats. The relative abundance of GSK3 fusion protein phosphorylated by the immunoprecipitated Akt was normalized to total Akt. Data are mean±SE (n = 3). abc: Groups without the same letter are significantly different (P<0.05). B. Soleus muscles from acetaminophen-treated (33T) and age-matched control (33C) animals were *ex vivo* incubated with insulin (+). Phosphorylation of GSK3α (Ser21), GSK3β (Ser9) and Akt (Ser473 and Thr308) after *ex vivo* insulin incubation were detected by immunoblotting. Muscle incubated with Krebs Henseleit buffer (−) was used as control of insulin response.

The *in vitro* kinase assay was performed to examine Akt functionality in aged muscle. The amount of GSK-3 fusion protein that was phosphorylated by immunoprecipitated Akt from very aged control animals was decreased by 32.8% and 34.6%, respectively, when compared to that observed using the 6-month or 27-month rats (P<0.05; Figure 4A). Acetaminophen administration increased the abundance of phosphorylated GSK-3 by 170.5% when compared to age-matched control (P<0.05).

To investigate the ability of aging muscle to respond to insulin, *ex vivo* muscle incubation studies were performed in the absence and presence of insulin. After *ex vivo* incubation with insulin, the phosphorylation of Akt and the Akt downstream proteins, GSK-3α and 3β, were increased in soleus muscles from acetaminophen-treated and age-matched control animals (Figure 4B). The phosphorylation levels of GSK3α and GSK3β were similar after insulin incubation between age-matched control and acetaminophen-treated rats, while there was higher phosphorylation of Akt Ser473 and Thr308 in the age-matched controls compared to that from acetaminophen-treated rats (Figure 4B).

### Akt dysfunction is associated with increases in Akt S-nitrosylation and iNOS

**Figure 5:**
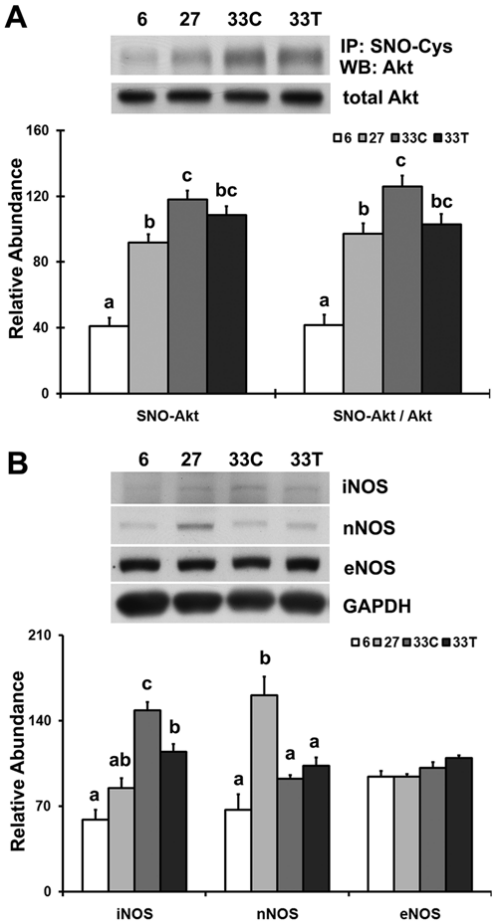
Akt dysfunction is associated with increases in iNOS and Akt S-nitrosylation. A. S-nitrosylated Akt in the soleus muscles of 6-, 27-, 33-month control (33C) and acetaminophen-treated (33T) rats was immunoprecipitated with anti-S-Nitroso-Cysteine (SNO-Cys) antibody and then detected by immunoblotting. B. iNOS, nNOS and eNOS proteins as detected by immunoblotting. Data are mean±SE (n = 4–6). abc: Groups without the same letter are significantly different (P<0.05).

The abundance of S-nitrosylated Akt protein was 188.6% and 28.6% higher in the soleus muscle of 33-month control rats than that observed in the 6- and 27-month animals, respectively (P<0.05; Figure 5A). After 6 months of acetaminophen treatment, S-nitrosylated Akt abundance was restored to that of 27-month animals (P>0.05). The ratio of S-nitrosylated Akt/total Akt in the very aged soleus muscle was 203.2% and 29.6% higher than that found in 6- and 27-month animals, respectively (P<0.05), while acetaminophen treatment restored the ratio to that observed in 27-month rats (P>0.05).

In an effort to understand the underlying mechanism of increased S-nitrosylated Akt in aging, we next examined the expression of nitric oxide synthase (NOS), an enzyme that *in vivo* has been shown to catalyze the production of nitric oxide. Compared to the 6- and 27-month animals, inducible NOS (iNOS) levels were higher in the soleus muscle of very aged control rats (151.3% and 74.7%, respectively; P<0.05; Figure 5B). After 6 months of acetaminophen treatment iNOS levels was restored to that observed in 27-month rats (P>0.05). The expression of neuronal NOS (nNOS) and endothelial NOS (eNOS) were not different between acetaminophen-treated and age-matched animals (Figure 5B).

### Akt hyper-phosphorylation is associated with a loss of PTEN protein

**Figure 6:**
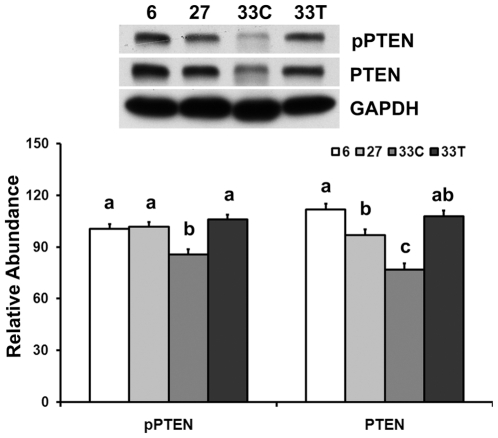
Akt hyper-phosphorylation is associated with a loss of PTEN protein. PTEN total protein and phosphorylation of PTEN at Ser380/Thr382/Thr383 in 6-, 27-, 33-month control (33C) and acetaminophen-treated (33T) rats as determined by immunoblotting. Data are mean±SE (n = 4–6). abc: Groups without the same letter are significantly different (P<0.05).

The abundance of phospho-PTEN (Ser380/Thr382/Thr383) and PTEN total protein in the very aged muscle were lower when compared to the adult rats (−14.9% and −31.0%, respectively; P<0.05; Figure 6). Chronic acetaminophen treatment increased the amount of phosphorylated PTEN (pPTEN) (Ser380/Thr382/Thr383) and PTEN protein by 23.9% and 39.9%, respectively, when compared to that in the age-matched control (P<0.05). Therefore, the ratio of pPTEN/PTEN in the acetaminophen-treated rats was 40.6% higher than that in the age-matched control (P<0.05).

### Akt dysfunction is associated with decreases in myosin and actin

**Figure 7:**
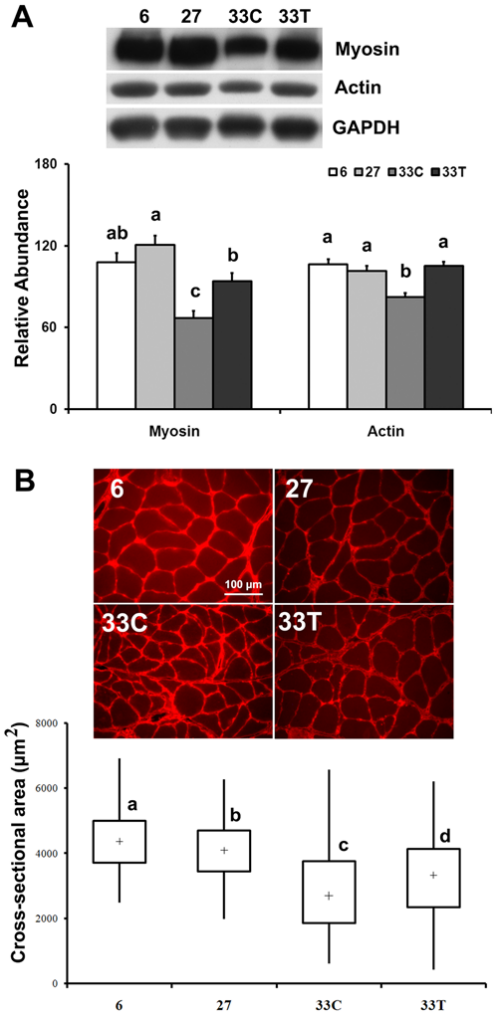
Akt dysfunction is associated with decreases in myosin, actin and muscle fiber cross-sectional area. A. Myosin and actin protein levels in 6-, 27-, 33-month control (33C) and acetaminophen-treated (33T) rats. Data are mean±SE (n = 4–6). B. Muscle fiber cross-sectional area (µm^2^/fiber). Upper panel: Representative images of dystrophin-stained soleus muscle section from 6-, 27-, 33C- and 33T rats. Lower panel: Vertical box and whisker plots showing the distribution of muscle fiber cross-sectional area: median values (+), the 25th and 75th percentile (the bottom and top of box, respectively), and the minimum and maximum (the bottom and top end of whisker, respectively). Number of fibers measured for 6-, 27-, 33C- and 33T-rats were 162, 207, 350 and 318, respectively. abcd: Groups without the same letter are significantly different (P<0.05).

Compared to that in 6- and 27-month animals, the amount of myosin (type I) was lower in the soleus of 33-month very aged rats (−38.0% and −44.6%, respectively; P<0.05; Figure 7A), while acetaminophen treatment restored myosin levels to a value equal to that found in the 6-month rats (P>0.05). The abundance of actin in 33-month control rats was 22.5% and 18.8% lower than that in 6- and 27-month animals, respectively (P<0.05; Figure 7A). Consistent with what we observed for myosin, chronic acetaminophen treatment restored actin protein to a level similar to that observed in 6-month animals (P>0.05).

### Akt dysfunction is associated with decreases of muscle fiber cross-sectional area

Soleus muscle fiber cross-sectional area was 7.6% and 35.4% lower in the 27- and 33-month control rats than that found in the 6-month rats, respectively (P<0.05; Figure 7B). Chronic acetaminophen treatment significantly increased the fiber cross-sectional area by 14.3% compared to the 33-month controls (P<0.05).

### Akt dysfunction is associated with increases in myocyte apoptosis

**Figure 8:**
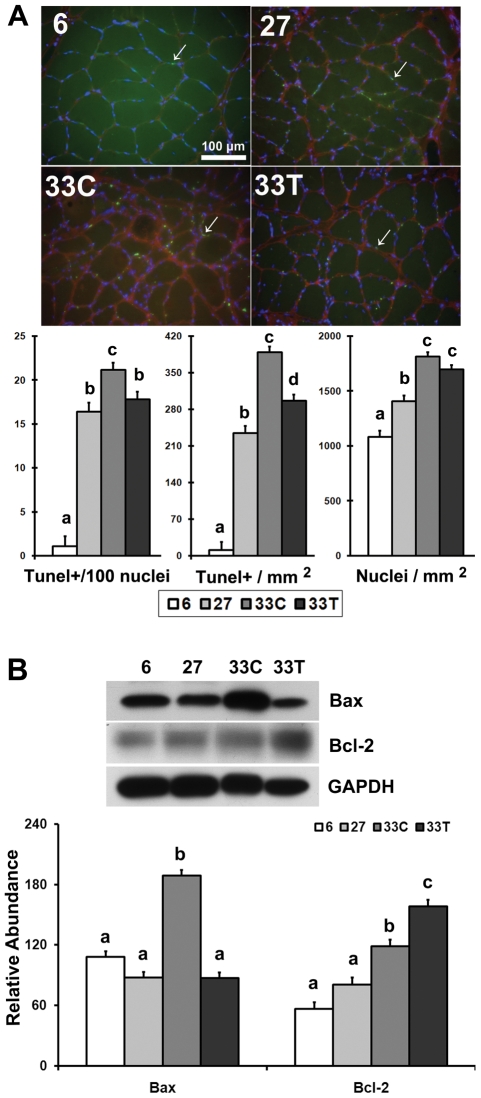
Akt dysregulation is associated with increases in myocyte apoptosis. A. DNA fragmentation in soleus muscles. Upper panel: Representative images of TUNEL-stained soleus muscle from 6-, 27-, 33-month control (33C) and acetaminophen-treated (33T) rats. Soleus sections were triple-stained with TUNEL (green), DAPI (blue) and dystrophin (red). Arrow indicates an example of apoptotic nuclei. Lower panel: Number of TUNEL-positive per 100 nuclei (Tunel+/100 nuclei), TUNEL-positive nuclei (Tunel+/mm^2^) and total nuclei (nuclei/mm^2^). Total nuclei counted for 6-, 27-, 33C- and 33T-rats were 1905, 3047, 6526 and 5425, respectively. B. Bax and Bcl-2 protein in the soleus of 6-, 27-, 33C- and 33T-rats as determined by immunoblotting. Data are mean±SE (n = 4–6). abcd: Groups without the same letter are significantly different (P<0.05).

Both the number of terminal deoxynucleotidyl transferase dUTP nick end labeling (TUNEL) positive nuclei per square millimeter and the number of TUNEL positive nuclei per 100 total nuclei were significantly increased with aging and were 3500.0% and 1899.0% higher than the levels found in 6-month animals, respectively (P<0.05; Figure 8A). After 6-months of acetaminophen treatment both the number and percentage of TUNEL positive nuclei were decreased by 23.7% and 15.8%, respectively (P<0.05). The number of total nuclei per square millimeter was 29.8% and 67.4% higher in 27- and 33-month control rats than the adult animals, respectively (P<0.05).

Compared to adult animals, Bax protein levels were 74.6% higher in the soleus muscle of very aged control rats (P<0.05; Figure 8B). Six months of acetaminophen treatment decreased Bax protein level by 53.8%, when compared to the age-matched control animals (P<0.05). The amount of Bcl-2 protein in very aged rats was 108.8% higher than that in adult animals, and acetaminophen treatment further increased Bcl-2 abundance by 33.4% when compared to age-matched control animals (P<0.05; Figure 8B). The ratio of Bax/Bcl-2 was 63.7% lower in the acetaminophen-treated rats than that observed in the age-matched control muscles (P<0.05).

## Discussion

Akt stands at the crossroads of several intracellular signaling pathways and plays important functions in regulating cellular survival, proliferation, transcription, and metabolism [5]–[9]. Using the F344BN aging rat model [24], we observed a hyper-phosphorylation of Akt (Ser473 and Thr308) in aging skeletal muscle. Paradoxically, this increased Akt phosphorylation is associated with lower Akt kinase activity, diminished response to insulin, higher levels of myocyte apoptosis, lower abundance of contractile proteins myosin and actin, and a smaller muscle fiber cross-sectional area (atrophy). Further, we demonstrate that acetaminophen administration functions to prevent age-associated Akt hyper-phosphorylation and dysfunction in aging muscle by decreasing S-nitrosylated Akt and restoring PTEN protein levels.

Activation of Akt requires the phosphorylation of Ser473 within the carboxyl-terminal hydrophobic motif, which primarily facilitates the phosphorylation of Thr308 in the activation loop to fully activate Akt kinase activity [12]. In the present study, phosphorylation of Ser473 and Thr308 were found to be higher in the soleus of very aged rats, which theoretically indicates a higher Akt kinase activity. Interestingly, these data do not appear to predict down-stream Akt signaling since the phosphorylation level of mTOR (Ser2448) [9] (Figure 2A), tuberous sclerosis complex-2 (TSC2) and forkhead box O1 (FoxO1) (data not shown) was lower in the very aged muscle. Similarly, the abundance of upstream Akt molecules, IR-β and PI3K [12], [16], were also lower in the very aged muscle. Additionally, PDK1 total protein, a kinase that is thought to phosphorylate Akt at Thr308 [10], and the phosphorylated (active) form of PDK1(Ser241) [25], were also lower in the very aged muscle. These results indicate that hyper-phosphorylation of Akt in aged muscle may be not directly related to the levels or phosphorylation of its putative upstream regulators. In an effort to understand the potential physiological significance of these findings, two important experiments, one *in vitro*, the other *ex vivo*, were performed. Using the *in vitro* Akt kinase activity assay we demonstrated that less GSK-3 fusion protein was phosphorylated by per unit of immunoprecipitated Akt obtained from very aged muscle. This finding demonstrates that Akt kinase activity is decreased with aging (Figure 4A). Interestingly, this decrease in activity occurs even though the amount of Akt phosphorylation (Ser473 and Thr308) is actually increased in the aged muscle. Using an *ex vivo* approach resulted in a similar conclusion as the aged control muscles required higher levels of Akt phosphorylation (both Ser474 and Thr308) to phosphorylate similar amounts of GSK3α and GSK3β compared to the acetaminophen-treated rats (Figure 4B). Taken together, these two different sets of experiments both suggest that aging in the F344BN soleus is associated with a mismatch between Akt phosphorylation and Akt kinase activity.

As a key molecule promoting anabolism and cellular survival, it is likely that age-associated Akt dysregulation may directly contribute to the deterioration of cellular metabolism and physiological function. In our study, the abundance of the contractile proteins, myosin and actin, were significantly decreased with aging (Figure 7A). Changes in actin and myosin, were in turn, associated with a decrease in mTOR, a key regulator of protein translation [7], [9]. It is possible that these decreases in myosin and actin contribute to the impairment of muscle function commonly observed with aging [2], [6], [26]. In addition to these losses in contractile proteins, we also observed a loss of myocyte size, a dramatic increase in the abundance of Bax, a shift in the Bax/Bcl-2 ratio and an increase in the number of myocytes undergoing apopotosis (Figures 7B, 8A and 8B). Interestingly, the restoration of Akt kinase activity by chronic acetaminophen intervention was associated with increases in the amount of contractile proteins and myocyte size, and a decrease in the incidence of myocyte apoptosis (Figures 7 and 8). Taken together these data suggest that an impairment in Akt signaling might be related to the development of age-related metabolic and functional disorders in skeletal muscle and importantly, that the re-establishment of Akt functionality may be a useful strategy for diminishing the deleterious effects of aging on muscle structure and function.

As an important signaling molecule involved in many physiological processes, nitric oxide regulates protein structure and function by interacting with cysteine sulfhydryls and inhibiting the formation of disulfide bonds [15]–[17], [27]. Increases in the amount of cellular S-nitrosylated proteins have been shown to be associated with the disruption of protein structure along with increases in tissue damage and mortality [17], [27]. S-nitrosylated proteins, including Akt, are involved in pathogenesis of insulin resistance [15], [16]. We found that both the abundance of S-nitrosylated Akt and ratio of S-nitrosylated Akt/total Akt were substantially increased with aging (Figure 5A). These increases occurred concomitant to increases in the amount of iNOS, an enzyme that catalyzes the production of nitric oxide needed for the S-nitrosylation reaction [18], [19]. Although it is unclear why aging might increase iNOS levels, other reports have suggested that increased extracellular glucose levels can induce iNOS expression [19], [20]. A previous study by our laboratory demonstrated that age-associated hyperglycemia and decreases in muscle glucose transporter-4 (Glut4) can be reversed by acetaminophen intervention [23], while other work using cultured cell has shown that acetaminophen can directly inhibit NO production and iNOS expression through its ability to diminish NF-kappaB binding to the iNOS gene promoter [28]. Consistent with these findings, we show that age-associated increases in iNOS expression were reversed after acetaminophen treatment, and further that this decrease in iNOS expression coincided with decreases in the amount of S-nitrosylated Akt (Figure 5A). As expected, this decrease in S-nitrosylated Akt was found to parallel the normalization of Akt phosphorylation and increases in Akt kinase activity. As such, it is likely that the S-nitrosylation of Akt is involved in contributing to age-associated Akt dysfunction. Unlike that observed for iNOS, acetaminophen did not appear to affect the regulation of nNOS or eNOS expression (Figure 5B) suggesting perhaps that the normalization of Akt S-nitrosylation by acetaminophen may be dependent on the reduced expression of iNOS. Why acetaminophen may target iNOS instead of other NOS isoforms is currently unclear. Further studies designed to examine the effects of aging on individual cysteine residues within the Akt protein will no doubt be useful in furthering our understanding on how S-nitrosylation might compromise Akt kinase activity.

The phosphatidylinositol 3′-phosphatase PTEN is considered a key negative regulator of Akt signaling [13], [14]. PTEN catalyzes phosphatidylinositol 3,4,5-trisphosphate (PIP3), a key mediator of PI3K activity, into phosphatidylinositol 4,5-trisphosphate (PIP2), resulting in the attenuation of phosphorylation (activation) of Akt [13], [14]. It has been reported that the phosphorylation of Thr308 of Akt, but not Ser473, is regulated by PTEN in adipocytes [13]. In present study, the abundance of pAkt-Ser473 was higher in muscles obtained from both the 33-month control and acetaminophen-treated rats, while pAkt-Thr308 was dramatically increased in control rats. These results are consistent with the alterations in PTEN protein levels and suggest that PTEN may regulate the phosphorylation of Akt Thr308 in skeletal muscle using a mechanism similar to that previously observed in adipocytes [13]. Interestingly, 6 months of acetaminophen intervention restored PTEN protein levels similar to that found in 6- and 27-month rats. This increase in PTEN protein appeared to parallel decreases in the amount of Akt-Thr308 phosphorylation which support the notion that a loss of PTEN protein with aging may contribute to the hyper-phosphorylation of Akt, and that acetaminophen intervention may function in reducing Akt phosphorylation by increasing PTEN levels.

Why aging decreases PTEN protein levels is not clear. It is thought that high glucose levels can result in decreased PTEN expression and decreases in PTEN phosphatase activity [14]. Our previous study found that the aging-associated hyperglycemia can be reversed by acetaminophen intervention [23]. It is possible that the normalization of blood glucose by acetaminophen may contribute to the increased PTEN expression. It has also been documented that phosphorylation of the C-terminal tail of PTEN decreases the degradation of PTEN protein by increasing its stability [29]. Our data also show that pPTEN levels were decreased with aging and that acetaminophen treatment restored pPTEN comparable to that observed in 6- and 27-month rats (Figure 6). Therefore, aging-associated hyperglycemia and decreases in pPTEN level may result in decreased PTEN protein levels, which could act to increase the phosphorylation of Akt at Thr308 in aging muscle. Whether changes in PTEN expression alone or if the presence of other factors is required to explain the effects of aging on Akt expression and phosphorylation will require further investigation.

**Figure 9:**
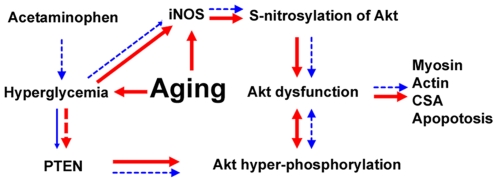
Proposed mechanism of age-associated Akt dysfunction and the effects of acetaminophen intervention. Red color: Aging is associated with increases in iNOS (dependent and/or independent on aging-associated hyperglycemia) and Akt S-Nitrosylation, leading to Akt dysfunction. Akt dysfunction is associated with increases in myocyte apoptosis, decreased myocyte cross-sectional area (CSA) and decreased expression of myosin and actin. Hyper-phosphorylation of Akt may be an important compensatory response to counteract Akt dysfunction with aging and may be related to an age-associated loss of PTEN protein. Blue color: Acetaminophen administration prevents aging-associated hyperglycemia, resulting in the attenuation of age-associated increases in iNOS and Akt S-Nitrosylation, which act to restore Akt kinase activity, decrease apoptosis and attenuate age-associated decreases in myocyte size, myosin and actin. Normalization of blood glucose levels by acetaminophen helps to increase PTEN expression. Increased PTEN attenuates age-associated hyper-phosphorylation of Akt, which may act to restore Akt functionality. Solid lines represent increase or activation, while square dotted lines represent decrease or inhibition.

In summary, age-associated decreases in muscle Akt kinase function may be related to increased muscle apoptosis and atrophy, and decreases in myosin and actin expression (Figure 9). Chronic acetaminophen treatment at a therapeutic dosage is able to restore the kinase activity of Akt in advancing age, which may be due to decreases in iNOS and diminished S-nitrosylation of Akt. The data of the present study show that a loss of PTEN protein in aging may contribute to the increased phosphorylation of Akt, and that acetaminophen intervention can restore both PTEN and pPTEN levels. This report provides evidence that Akt may be a key target molecule for interventions designed to attenuate the effects of aging on skeletal muscle and that acetaminophen may be useful for the treatment of age-related muscle disorders.

## Materials and Methods

### Materials

Primary antibodies against Akt (#9272 and #2920), phospho-Akt (Thr308) (pAkt-Thr308, #9275), phospho-Akt (Ser473) (pAkt-Ser473, #9271), GAPDH (#2118), GSK3α (#9338), GSK3β (#9315), phospho-GSK3α/β (Ser21/9) (#9331), IR-β (#3025), mTOR (#2972), phospho-mTOR (Ser2448)(pmTOR, #2971), PI3K-p85 (#4257), PTEN (#9552) and phospho-PTEN (Ser380/Thr382/383) (pPTEN, #9554), PDK1 (#3062), phospho-PDK1 (Ser241)(pPDK1, #3438), nNOS (#4231), eNOS (#9572), secondary antibodies conjugated with horseradish peroxidase (HRP) (anti-rabbit (#7074) or anti-mouse (#7076)) and nonradioactive Akt kinase assay kit (#9840) were purchased from Cell Signaling Technology (Beverly, MA). Primary antibodies against Bcl-2 (sc-7382) and Bax (sc-493) were from Santa Cruz Biotechnology (Santa Cruz, CA). Dystrophin antibody (NCL-DYS2) was from Novocastra Vector Laboratories (Burlingame, CA). Actin antibody (MCA1906) was from Antibodies Direct (Raleigh, NC). S-Nitroso-Cysteine (SNO-Cys, #N5411) and myosin (M8421) primary antibodies, and acetaminophen (#A5000) were from Sigma-Aldrich, Inc. (St. Louis, MO). Human recombinant insulin was from SAFC Biosciences (Lenexa, KS). The *in situ* cell death detection kit was from Roche Diagnostics (Mannheim, Germany). VECTASHIELD HardSet Mounting Medium with DAPI was from Vector Laboratories (Burlingame, CA). Pierce Tissue Protein Extraction Reagent (T-PER) and Pierce 660nm protein assay reagent (#22660) were from Thermo Fisher Scientific Inc. (Rockford, IL). The PAGEr Gold Precast gel (10%) was from Lonza (Rockland, ME). The Amersham ECL™ Western Blotting reagent (RPN 2106) was from GE Healthcare Bio-Sciences Corp. (Piscataway, NJ).

### Animals

The F344BN rats (National Institute on Aging, Bethesda, MD) were housed in an Association for Assessment and Accreditation of Laboratory Animal Care International (AAALAC) approved vivarium with a 12∶12 h light/dark cycle at 22±2°C. Food (LabDiet 5001, PMI Nutrition International, LLC, Brentwood, MO) and water were provided *ad libitum*. All procedures were approved by the Marshall University Institutional Animal Care and Use Committee, and the “Principles of laboratory animal care” (NIH publication No. 86-23, revised 1985) were followed.

### Acetaminophen treatment and tissue collection

Twenty seven months old F344BN rats were given acetaminophen daily (30 mg/kg body weight/day) for 6 months in their drinking water. Age-matched rats were maintained as controls. Soleus muscles were removed from anesthetized animals, frozen in liquid nitrogen, and stored at − 80°C.

### *Ex vivo* muscle incubation with insulin

Two strips of muscle were isolated from each soleus muscle as previously described [30]. To avoid tissue hypoxia that may compromise structure and function of muscle, *ex vivo* muscle incubation were performed at 25°C in Krebs Henseleit buffer (KHB, pH 7.4) containing 5.5 mM glucose, 2 mM pyruvate, 5 mM HEPES and 0.1% bovine serum albumin, and equilibrated with 95% O_2_ and 5% CO_2_ as outlined elsewhere [31]. Muscles were mounted to plastic plates to keep them at their *in situ* resting length. After pre-incubation in KHB for 15 min, muscles were incubated in KHB for either another 30 min (control), or in KHB containing human recombinant insulin (100 µU/mL) for 30 min. Muscle samples were then washed with KHB, immediately frozen in liquid nitrogen, and stored at − 80°C.

### *In vitro* Akt kinase activity assay

To assess Akt kinase activity, total Akt protein was immunoprecipitated with anti-Akt antibody (#2920) overnight at 4°C, and then the kinase activity assay was performed using a nonradioactive Akt kinase assay kit as outlined by the manufacturer (#9840). Briefly, after washing with cell lysis buffer and kinase buffer, the immunoprecipitates were incubated with a GSK-3 fusion protein substrate and ATP for 30 min at 30°C. The phosphorylation of the GSK-3 fusion protein at Ser21/Ser9 by the immunoprecipitated Akt was detected by immunoblotting. The immunoprecipitated Akt protein was detected using anti-Akt antibody (#9272). The abundance of p-GSK-3 was normalized to the amount of total Akt.

### Analysis of S-nitrosylated Akt

To detect S-nitrosylated Akt, soleus lysates were immunoprecipitated by anti-S-Nitroso-Cysteine antibody overnight at 4°C. Akt in the immunoprecipitates were detected by immunoblotting.

### Immunoblotting analysis

Protein samples was prepared, separated on a 10% PAGEr Gold Precast gel, and then transferred to nitrocellulose membranes as previously described [23]. After incubated with primary antibody overnight at 4°C and secondary antibody for 1 h at room temperature, target proteins were visualized following reaction with Amersham ECL reagent. Target protein levels were quantified by an AlphaEaseFC image analysis software (Alpha Innotech, San Leandro, CA) and normalized to GAPDH.

### TUNEL, Dystrophin and DAPI triple-staining

DNA fragmentation associated with apoptosis was detected by TUNEL, using a Roche *in situ* cell death detection kit according to manufacturer instructions. Soleus sections were blocked with 3% BSA and incubated with anti-dystrophin antibody at the dilution of 1∶200 to visualize the cell membrane. Nuclei were co-stained using DAPI in a VECTASHIELD HardSet Mounting Medium. Sections were visualized under an Olympus BX51 microscope equipped with Olympus WH 10×widefield eyepieces and Olympus UPlanF1 40×/0.75 objective lens.

### Quantification of muscle fiber cross-sectional area

Muscle fiber cross-sectional area was determined by tracing the outline of dystrophin-stained fibers using the ImageJ program (http://rsb.info.nih.gov/ij/). The distribution of muscle fiber cross-sectional area was plotted with vertical box and whisker plots, and multiple comparisons were performed to determine differences of means between groups (See “Data analysis”).

### Data analysis

Results are presented as mean±SE. The effects of age and acetaminophen treatment were analyzed using the GLM procedure (SAS 9.1 for Windows, SAS Institute Inc., Cary, NC). Means were calculated by the LSMEANS procedure and multiple comparisons were performed using the Tukey-Kramer test to determine differences between groups. Values of P<0.05 were considered to be statistically significant.
